# A guide to translation of research results from model organisms to human

**DOI:** 10.1186/s13059-016-1026-9

**Published:** 2016-07-26

**Authors:** Ross C. Hardison

**Affiliations:** Department of Biochemistry and Molecular Biology, Huck Institute for Genome Sciences, The Pennsylvania State University, University Park, PA 16802 USA

## Abstract

A new study helps resolve a controversy about determinants of gene expression variability and might facilitate the effective translation of research results across species.

## Introduction

The utility of experiments in model organisms such as mice to provide insights into human health and disease depends on the similarity of the fundamental physiological, cellular, and molecular processes across species. For example, one might expect that gene expression profiles of liver between mouse and human would be more similar than expression profiles between mouse heart and liver. However, recent papers have reached different conclusions as to whether expression profiles were more different between organs or between species. A paper from Breschi and colleagues [1] in this issue of *Genome Biology* provides refreshing insight into this controversy. The authors show that the expression patterns for some genes are dominated by variance between organs, whereas the patterns for others are dominated by variance between species. Classifying each gene by the main source of its variance in expression brings out important functional differences. Importantly, the tissue-variable genes (TVGs) are enriched for genetic variants associated with complex traits, including disease susceptibility, whereas the species-variable genes (SVGs) are less enriched.

## Is it not obvious that tissue-specific gene expression would dominate over differences between related species?

Even before the advent of sequence-based molecular genetics, annealing reactions between RNA and DNA showed that a population of RNA was specific to individual tissues or organs. This tissue-specific gene expression could explain the differences between tissues, and it laid the foundation for the guiding principle of developmental biology—differential gene expression determines (in large part) the distinctive properties of each cell type [2]. Research in the current era of sequence-based molecular biology confirmed this guiding principle, and for decades we have learned much about mechanisms governing differential gene expression. Thus, it seems like an obvious extrapolation to think that in species with similar body plans and overall physiology, such as human and mouse, comparisons of the transcriptional profile across all genes in homologous organs would reveal greater similarity between the homologous organs across species than between different organs in the same species. Specifically, in a comparison of transcriptomes of multiple homologous organs across species, the samples should cluster by organ, not by species.

## After some dispute, interspecies clustering by tissues is robust

The published results have been more complicated than expected. Multiple reports examining microarray hybridization (e.g., [3]) or RNA-seq data (e.g., [4–6]) showed the predicted clustering by organ. However, others showed the opposite—that is, clustering by species (e.g., [7]). To resolve these conflicting results, a meta-analysis of the RNA-seq data from four independent studies of transcriptomes across species and tissues, including the data from Lin and colleagues [7], was conducted. This recent study found a strong, robust clustering by tissue across species [8].

Breschi et al. [1] also re-examine the relationships among the global transcriptomes across organs and species. They do not limit their analysis to visualization of relationships by dimensional reduction techniques employed in many earlier papers, but instead develop a quantitative approach to assess the contributions of species or organ to overall transcriptome relationships. From the pairwise correlations among transcriptomes, they generate a network of nodes (transcriptome of an organ from a given species) and edges (significant correlations between transcriptomes) and then analyze whether the modularity of that network is determined more by species relationships or organ relationships. The quantitative modularity analysis shows that variation in expression among organs dominates over variance among species.

These several studies and re-analyses revealed confounding and complicating factors that impact the interpretation of the results. Issues with study design and the need to avoid batch effects have been emphasized previously [9]. In addition, it is important to realize that differences in the sets of tissues examined and phylogenetic distances among the species, as well as mode of analysis (e.g., pairwise distances versus dimensional reduction approaches), can affect the clustering patterns [1, 8].

## Embrace the diversity in variation of expression patterns

The analyses described so far treat the transcriptome of an organ from each species monolithically and the resulting global relationships are highly informative. However, the results also reveal a subset of genes with significantly altered expression patterns across species [4], suggesting that this subset of genes could contribute to species-specific or lineage-specific features. Of course, other genes show patterns of expression in different tissues that are well preserved across species, reflecting a conserved regulatory program for this subset of genes [3].

**Fig. 1:**
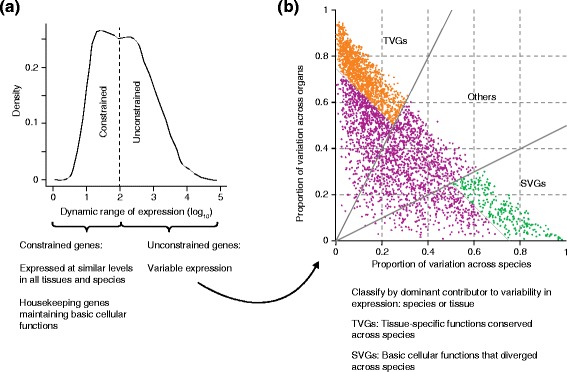
Classifying individual genes by expression variability across species and organs. **a** Genes are separated by their dynamic range of expression (across all organs or tissues in all species) into constrained or unconstrained categories. Redrawn from reference [10]. **b** The unconstrained genes are classified by the extent to which variation across species or across organs contributes to overall expression variation for each gene (represented as a *data point*). Genes for which at least 75 % of the expression could be attributed to organ or species (*line with negative slope*) were further divided into tissue-variable genes (*TVGs*, *orange*) and species-variable genes (*SVGs*, *green*) using a two-fold threshold for the greater contribution of organ than species for TVGs and vice versa for SVGs (*lines with positive slopes*). The remaining unconstrained genes were classified as “*Others*” (*purple*). Redrawn from reference [1]

Given these distinct subsets of genes, Breschi and colleagues [1] focus on the determinants of expression variation for *individual* genes. They classified genes by the extent to which their expression patterns were dominated by species differences or tissue differences, using data from a previous RNA-seq study [5] that included six organs from each of seven species, ranging from human to chicken. Breschi et al. developed linear models to quantify, for each gene, the contribution of variation across organs and across species. Using the results of these analyses, they divided 6283 orthologous genes into four informative categories (Fig. 1). A large number of genes (2661), termed “constrained”, show little variation in expression across either tissue or species [10]. The 3622 “unconstrained” genes were placed into three classes (Fig. 1b). Genes for which expression variation could largely be attributed to organ or species were further divided into tissue-variable genes (1245 TVGs) and species-variable genes (268 SVGs) using arbitrary, but stringent, thresholds. The remaining 2109 were classified as “others”.

The sets of SVGs and TVGs differ in striking ways. The SVGs are enriched in gene ontology terms indicative of basic metabolic processes (“housekeeping” functions), whereas TVGs are enriched in tissue-specific functions such as neurogenesis. While the “other” genes do not pass the stringent thresholds for contribution to expression variation, they should not be ignored. They are enriched for ontology terms of processes often implicated in adaptive evolution, such as the immune response.

## Translation of research results from mouse to human

This gene-centric view of expression variation provides a path for interpreting results from studies in animal models to infer implications for human health and disease. Rather than insisting on a global ability to translate from mouse studies to human inference, one can take a more nuanced and accurate approach that embraces the diversity of courses of expression variation. The TVGs are differentially expressed across tissues and have highly similar patterns of expression across species. Thus, results about the roles or regulation of such genes from genetic and biochemical studies in mouse should translate readily to inferences about their roles in human and validation of those inferences in human should be straightforward. Breschi and colleagues show that trait-associated variants discovered in genome-wide association and other studies are enriched in TVGs, further emphasizing the importance of this set of genes for translation of research inferences to human. The large class of constrained genes, which have limited variation in expression across both tissue and species, might also be straightforward for translation of results from mouse to human.

By contrast, if research results from mouse systems reveal a function for an SVG, then the inference that it has the same function in human needs to be considered with caution. The human ortholog of the gene investigated in mouse does have a different expression pattern and thus validation experiments in human cell systems might need to be more extensive than for TVGs. Note that the fact that a gene is in the SVG category does not necessarily render it useless as a potential mouse model, but it does raise the bar for validation of inferences in human. Also, further investigation might establish a species-specific role for this SVG.

Genes that are unconstrained in expression patterns but do not pass the stringent thresholds to be included in the TVG or SVG sets could also be implicated in particular physiological processes in model systems. The analyses from Breschi and colleagues can also provide some guidance for ease or difficulty in translation of results to human. The estimated contribution of species or organ to expression variation is provided for each orthologous gene, regardless of category, and one can use this information to infer how similar the expression patterns are between mouse and human. Indeed, while threshold-based assignments of genes to the major categories helps clarify the diversity of patterns of expression variation, the quantitative estimates of dynamic range of expression and sources of expression variability, regardless of categorical assignment, could prove to be one of the most useful products of this research. Such estimates should be considered as informative attributes of genes, in addition to the more familiar features such as gene ontology terms and phylogenetic sequence conservation, that can help guide translation of research results from model organisms to human.

## Abbreviations

SVG, species-variable gene; TVG, tissue-variable gene
